# Alterations in the Microbiomes and Metabolic Profiles of the Ileal Between the Hu Sheep and East Friesian Sheep

**DOI:** 10.3390/ijms252413267

**Published:** 2024-12-10

**Authors:** Wenna Yao, Yue Zhao, Shuo Yan, Huimin Zhang, Teligun Bao, Siqin Bao, Xihe Li, Yongli Song

**Affiliations:** 1 Research Center for Animal Genetic Resources of Mongolia Plateau, College of Life Sciences, Inner Mongolia University, Hohhot 010020, China; yaowenna024@163.com (W.Y.); zhao_yue0230@sina.com (Y.Z.); yanshuo202305@163.com (S.Y.); zhanghuimin202404@163.com (H.Z.); 13947356727@163.com (T.B.); baosq@imu.edu.cn (S.B.); 2 The State Key Laboratory of Reproductive Regulation and Breeding of Grassland Livestock, College of Life Sciences, Inner Mongolia University, Hohhot 010020, China

**Keywords:** Hu sheep, East Friesian sheep, disease-resistant phenotype, ileal microorganisms, metabolites

## Abstract

The East Friesian sheep is a dairy breed known for its high fertility and high milk production and is currently one of the best dairy sheep breeds in the world. This breed is known to have a poor disease-resistant phenotype compared to Hu sheep. Gut microbiota and metabolites play a role in host disease resistance. The intestinal bacterial microbiota is essential for maintaining the health of sheep and ensuring their productive potential, and it may also help explain disease-resistant phenotypic differences related to breeds. However, the ileum microbiota and metabolite profiles of Hu sheep and East Friesian sheep have remained poorly characterized. The ileal is a significant organ in the intestinal tract, and most nutrients and minerals in food are absorbed through the small intestine. It is necessary to understand the composition of both species’ ileal microbiota and metabolites using the same feeding conditions. Therefore, studying the differences in the ileal microorganisms between breeds is essential to decipher the mechanisms behind these differences and identify microorganisms that influence the disease-resistant phenotype drive of ruminants. Due to the poor disease-resistant phenotype in sheep during the weaning period, with diarrhea and other diseases most likely to occur, we selected dairy sheep that were just two months old and had recently been weaned. This study comprehensively examined differences between the ileal microbiota in a large cohort of two breeds of sheep, including six Hu sheep and six East Friesian sheep. Using 16S rRNA and non-targeted metabolomics analysis, we determined that the Hu sheep had more microorganisms, including *Lactobacillus*, *Bifidobacterium*, *Streptococcus*, and *Limmosilactobacillus,* and more metabolites, including 2,7-Dihydroxy-5-methyl-1-naphthoic acid, Leu-Pro-Glu-Phe-Tyr, dodecanoic acid, Ala-Gln-Phe-Ile-Met, and Ala-Gln-Glu-Val-His, compared to the EF sheep group. Moreover, the Hu sheep were significantly enriched in amino acid biosynthesis, fatty acid metabolites, and bile secretion compared to the EF sheep groups, which may have been the main driver of the observed differences in disease-resistant phenotypes between the Hu sheep and East Friesian sheep. In addition, we hypothesized that there may be multiple beneficial microbes and metabolites that modulate the immune response and ultimately affect disease resistance. Therefore, these findings provide insights into the mechanisms underlying disease-resistant phenotype in sheep and may provide useful information for optimizing the composition of the ileal bacterial microbiota in sheep.

## 1. Introduction

The gut microbiota has been called the second human genome [1]. The intestinal tract of ruminants has a complex microecosystem that plays a crucial role in feed degradation, metabolism, tissue development, and host immune regulation [2]. Each region in the gut contains a different microbial community [3]. Even within the same species, different microbial communities in the gut exhibit great diversity, and current data suggest a relationship between host characteristics and gut bacteria [4]. The gut microbiota changes differently in different stages, which is the cause or result of changes in host health and disease status [5]. In ruminants, in addition to the rumen, the study of the small intestine’s microbes also plays a crucial role in understanding the microbiota of ruminants, especially the ileum [6]. The ileum is the intestinal contents between the thin bacteria in the proximal small intestine and the large colon and the proximal small intestine and the colon. The number of bacteria is 10^7^~10^8^ CFU/mL, and the types of bacteria are more than that in the proximal small intestine, mainly aerobic bacteria or facultal anaerobic bacteria, such as *Bacillus*, *Clostridium*, *Enterobacter*, and *Lactobacillus* [7]. The ileum can affect the nutrition and health of the host by enhancing metabolic capacity, defending against pathogens, and regulating gastrointestinal development and the immune system, and the ileum microbes play a major role in this process [8].

The genetic background and environmental factors influence the assembly of microbial communities formed by natural selection and co-evolution with hosts [9]. Studies on different animal species, such as Meishan and Yorkshire pigs and East Friesian sheep and Hu sheep, have shown that differences in gut microbiome composition are significantly associated with disease resistance [10]. In Meishan pigs, the greater stability of the gut flora and intestinal homeostasis contribute to higher disease resistance, as confirmed by models using dextran sulfate sodium (DSS) to induce inflammation [11,12]. Under the same controlled diet conditions, buffaloes showed better fiber digestion compared to healthy Holstein cattle, which could be attributed to the influence of gut microbiome regulation [13]. The different abundance of intestinal microbes in healthy yaks and cattle are mainly influenced by the host’s adaptation to extreme environments [8]. *Bacillus subtilis* could protect mice against Salmonella typhimurium infection by promoting stem cell differentiation to improve its disease resistance in the ileum [14]. The gut microbiota has also been reported to aid in metabolic responses to facilitate the clearance of toxic reactive metabolites [15]. Metabolites include tangible compounds produced during metabolic processes that are both the substrates and results of metabolic reactions [16]. It has been proposed that small molecules produced by the co-metabolism of the host and microbiome, such as amino acids, short-chain fatty acids, and bile acids, have important effects on host physiological function [17]. In ruminants, microbiota and metabolite interactions are primarily associated with gut-related diseases, including diarrhea, enterotoxemia, and inflammatory bowel disease (IBD) [16]. These diseases mainly influence mucous membranes, barrier function, nutrient absorption, energy storage, inflammation, and immune responses, ultimately impacting the overall health status of the host organism and host resistance [18]. This approach visually describes the molecular metabolic profile generated by the metabolism of individuals, organ tissues, and cellular entities, thereby establishing a link between metabolic pathways and underlying biological mechanisms [17].

The East Friesian sheep (EFS) is one of the most productive dairy sheep breeds that provide mutton and dairy products needed for daily life [19]. East Friesian sheep have been imported into many countries and are often used to improve the quality of their native sheep due to their good milk and meat-producing properties [20]. The annual consumption of mutton and sheep milk exceeds production levels in China [10]. Therefore, we need to increase production performance including increasing the production of mutton and sheep milk to meet the demand in livestock. The gut microbiota significantly affects the productivity of ruminants, and disease resistance is one of the important reasons for affecting productivity [21]. Hu sheep are characterized by fast growth, strong environmental adaptability, high-temperature- and cold-resistance, tolerance to rough feeding, suitability for large-scale captivity, etc., and most importantly, they have stronger disease resistance [22]. However, different breeds of sheep are subject to physiological and environmental influences during weaning that adversely affect their growth, development, and health, resulting in slow growth, enteritis, and increased mortality [23]. In production practice, we found that East Friesian sheep are more susceptible to diseases such as pneumonia, enteritis, and others compared to local breeds under the conditions of local breeding [24]. The costs associated with pneumonia, enteritis, and other diseases in the livestock sector, including deaths and lost productivity range from USD 10 million to USD 29 million each year, and the cost is increasing every year [10]. Disease resistance is one of the most important reasons and it can affect production performance. A growing body of research shows differences in disease resistance between different species of sheep [18].

Currently, most of the literature focuses on studying the gut microbiome of pigs and cattle. However, there are no studies on the ileal microbiota and metabolites of East Friesian sheep and Hu sheep, and most of the above studies have not explored the differences in the intestinal microbiome between different breeds. In this study, the gut microbiome and metabolomics were used to decipher the mechanism of heterogeneity in East Friesian sheep and Hu sheep, which we will demonstrate in the following sections. Assessing the differences in the ileal microbiota and metabolites between breeds enhances our understanding of how the ileal microbiome of different sheep breeds affects disease resistance. The findings of this study will provide valuable insights into the role of the ileal microbiome in sheep health and disease resistance, offering new perspectives for improving sheep farming practices.

## 2. Results

### 2.1. The Composition and Diversity of the Ileal Microbiota Between the Hu Sheep and East Friesian Sheep

**Figure 1:**
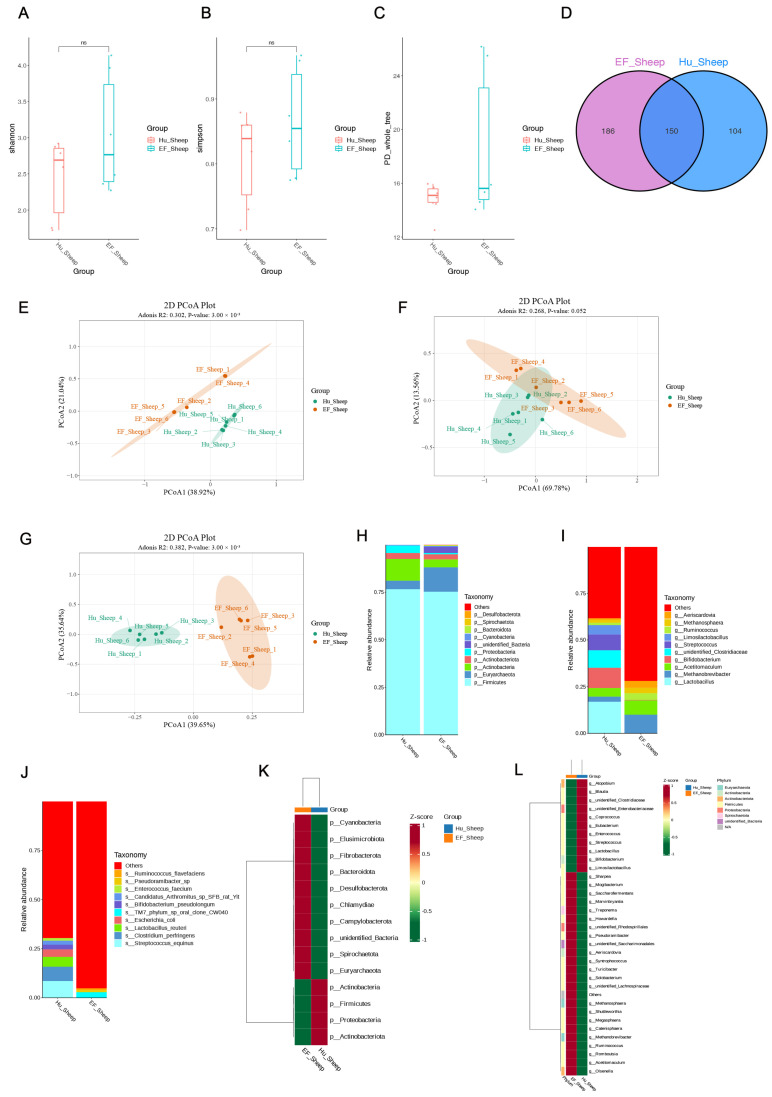
The composition and diversity of the ileal microbiota between the Hu sheep and East Friesian sheep. (**A**–**C**) The Shannon, Simpson, and PD-whole-tree indices of the gut microbiota. (**D**) A Venn diagram showing the common OTUs identified in the gut microbiota between the two groups. (**E**–**G**) The PCA scaling of ileum microorganisms. (**H**–**J**) The top 10 microbiota taxa at the phylum, genus, and species levels. (**K**,**L**) Hierarchical clustering heatmaps of all significantly differentially abundant gut microbiota between the two groups. Data are presented as the mean ± SEM. ns: *p* > 0.05.

Using the breed differences between Hu sheep and East Friesian sheep, we determined the ileal microbiota composition in the Hu sheep and EF sheep groups by 16S rRNA sequencing. To determine the relative abundance of species, we analyzed the Shannon, Simpson, and PD-whole-tree indices of α variation. Compared to the Hu sheep group, there was no significant difference in the Shannon, Simpson, and PD-whole-tree indices in the EF sheep group (*p* > 0.05) (Figure 1A–C). We determined the total microbial community in the two types of sheep, including 440 microorganisms common to both sheep, 186 microorganisms endemic to the EF sheep group, and 104 microorganisms endemic to the Hu sheep group (Figure 1D). Our results confirmed that the gut microbiota may vary between the two breeds of sheep. The similarity and β diversity between the microbial communities was assessed by PCA (principal component analysis), which represented that the total variations between the microbial communities were 38.92%, 69.78%, and 39.65%, respectively (Figure 1E–G). The results revealed that the intestinal flora of the EF sheep group were remarkably different from those of the Hu sheep group.

To further understand the microbial differences in the different breeds, we assessed the taxonomic composition of the ileal microbiota. We witnessed a major difference in the gut bacteria at the phylum, genus, and species levels in the sheep of different breeds. At the phylum level, the Hu sheep group revealed a higher relative abundance of *Firmicutes*, *Actinobacteria*, and *Actinobacteriota*, while *Euryarchaeota*, *Bacteroidota*, *unidentified_Bacteria* and *Spirochaetota* were revealed in a lower relative abundance compared to the EF sheep group (Figure 1H). At the genus level, the Hu sheep group revealed a higher relative abundance of *Lactobacillus*, *Bifidobacterium*, *Streptococcus*, and *Limosilactobacillus* and revealed a lower relative abundance of *Methanobrevibacter*, *Ruminococcus*, *Methanosphaera*, and *Aeriscardovia* than the EF sheep group (Figure 1I–L).

We found that beneficial gut microbes clustered in the Hu sheep group, while the abundance of these probiotics was reduced in the EF sheep group. Specifically, we found that the potential probiotics *Lactobacillus*, *Limosilactobacillus*, and *Bifidobacterium*, had a higher relative abundance in the Hu sheep group. In contrast, the abundance of these probiotics was lower, and potentially harmful bacteria such as *Bacteroides* and *unidentified bacteria* were clustered in larger numbers, in the EF sheep group. These harmful bacteria were concentrated in the East Friesian sheep and may play a role in fighting infection. This phenomenon may be one of the reasons for the difference in disease resistance phenotypes between the two breeds. However, the ileal of the Hu sheep was also enriched with potentially beneficial microorganisms, which may play a certain therapeutic and protective role in the gut [25]. *Lactobacillus* and *Bifidobacterium* can produce beneficial metabolites such as lactic acid (amino acid), break down and metabolize bile salts, inhibit proinflammatory mediators, and have a repairing and protective effect in the gut [7].

**Figure 2:**
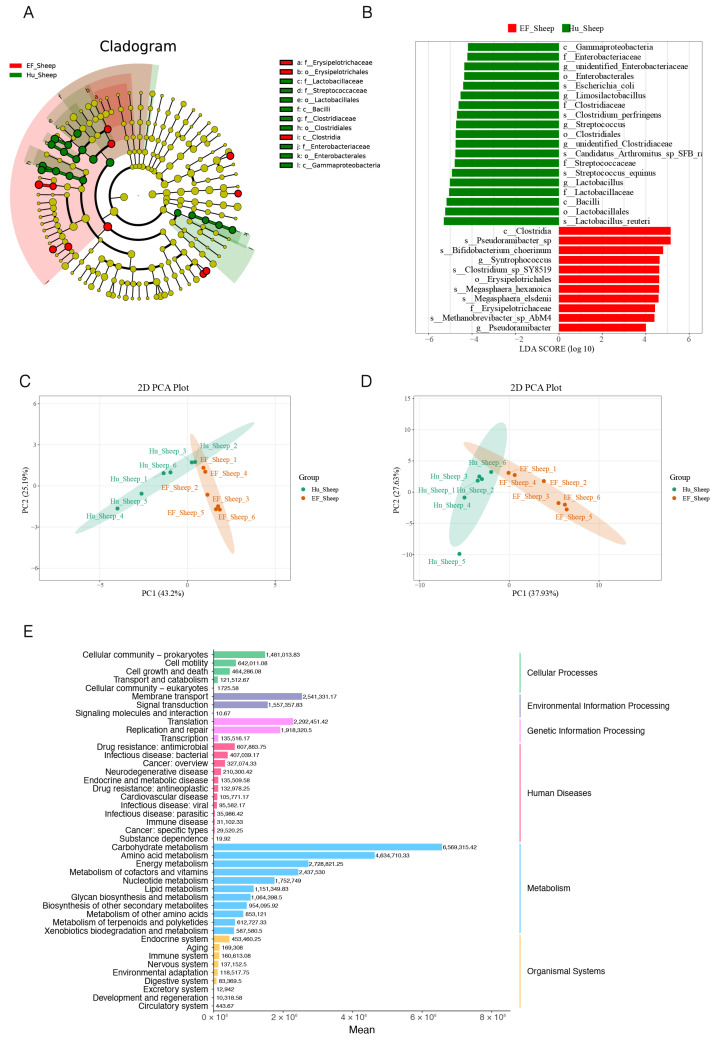
The composition and diversity of the ileal microbial community. A cladogram and LEfSe analysis of the gut microbiota between the Hu sheep and East Friesian sheep. (**A**,**B**) A cladogram and LDA score plot generated from the LEfSe of 16S rRNA gene amplification sequencing data (LDA score > 4; *p* < 0.05) in the Hu sheep versus the East Friesian sheep. (**C**,**D**) A demonstration of a PICRUSt2 (phylogenetic investigation of communities by reconstruction of unobserved states) functional annotation of the PCA results based on the ASV in the primary functional classification. (**E**) A PICRUSt2 secondary-classification-abundance histogram based on the ASV. Data are presented as the mean ± SEM.

Also, the LEFSe (linear discriminant analysis effect size) was employed to assess the relative abundance of microbes in the ileal region of the two breeds. To display the microbes with the most significant difference between the two breeds, we generated an evolutionary phylogenetic tree. Consistently, the outcomes revealed that *s_Lactobacillus_reuteri*, *o_Lactobacillales*, *c_Bacilli*, *f_Lactobacillaceae*, and *g_Lactobacillus* were the most differentially enriched in the Hu sheep group compared to the EF sheep group. Similarly, the outcomes indicated that *c_Clostridia*, *s_Pseudorami-bacter_sp*, *g_Syntrophococcus*, *s_Clostridium_sp_SY8-519*, and *o_Erysipelotrichales* were the most differentially enriched in the EF sheep group compared to the Hu sheep group (Figure 2A,B).Compared to the Hu sheep group, findings revealed that the EF sheep group varied from the other group because of the abundance of harmful bacteria. Next, we analyzed the microbial function of the PCA results and observed differences in the microbial function between the Hu sheep group and the EF sheep group (Figure 2C,D). Moreover, the main enrichment pathways were cell growth, death, transport, and catabolism; membrane transport; signaling molecules; infectious diseases, including viral and immune diseases; carbohydrate metabolism; amino acid metabolism; lipid metabolism; and the biosynthesis of other secondary metabolites in secondary functional classifications (Figure 2E). In summary, there was spatial heterogeneity in the bacterial flora in the intestinal tract, and biomarkers of specific microbes were identified between the varieties.

### 2.2. Differences in Metabolites in Ileum Between Hu Sheep and East Friesian Sheep

**Figure 3:**
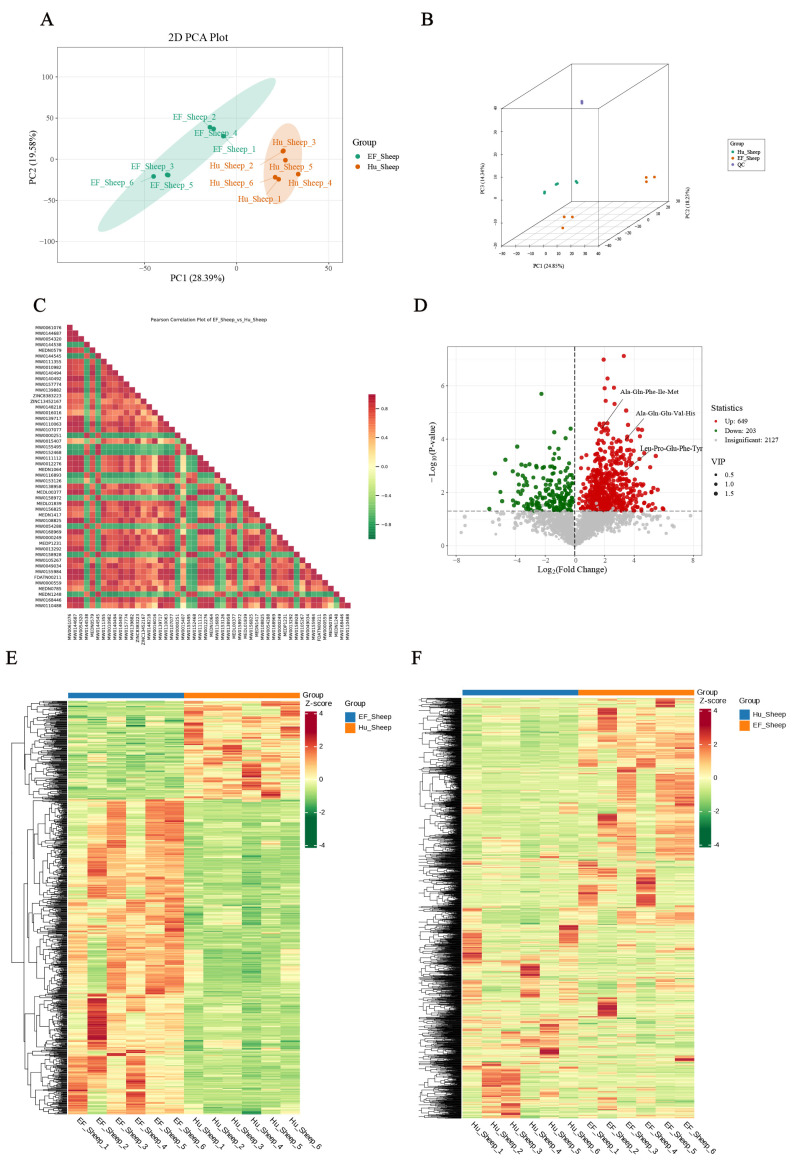
Differences in metabolite abundance in the intestine between the Hu sheep and East Friesian sheep. Changes in the intestinal metabolome. (**A**,**B**) A PCA plot of the ileum metabolites. (**C**) Correlations of the metabolites between the Hu sheep and East Friesian sheep. (**D**) A volcano plot of differential metabolites between the Hu sheep and East Friesian sheep. (**E**,**F**) Hierarchical clustering heatmaps of all significantly differentially abundant metabolites between the two groups.

Since microbes and metabolites are closely related, microorganisms can secrete multiple metabolites to impact the host. We evaluated two types of ileal contents by nontargeted metabolites. The PCA results indicated a significant variation between the breeds in the metabolites of the ileum contents (Figure 3A,B). A comparison of the metabolites exhibited a relationship between them (Figure 3C). There were 649 metabolites with upregulated expression and 203 metabolites with downregulated expression in the EF sheep group compared to Hu sheep group, including Ala-Gln-Phe-Ile-Met, Ala-Gln-Glu-Val-His, and Leu-Pro-Glu-Phe-Tyr (Figure 3D). A total of 2006 metabolites revealed a significant difference in the Hu sheep group compared to the EF sheep group (Figure 3E,F).

**Figure 4:**
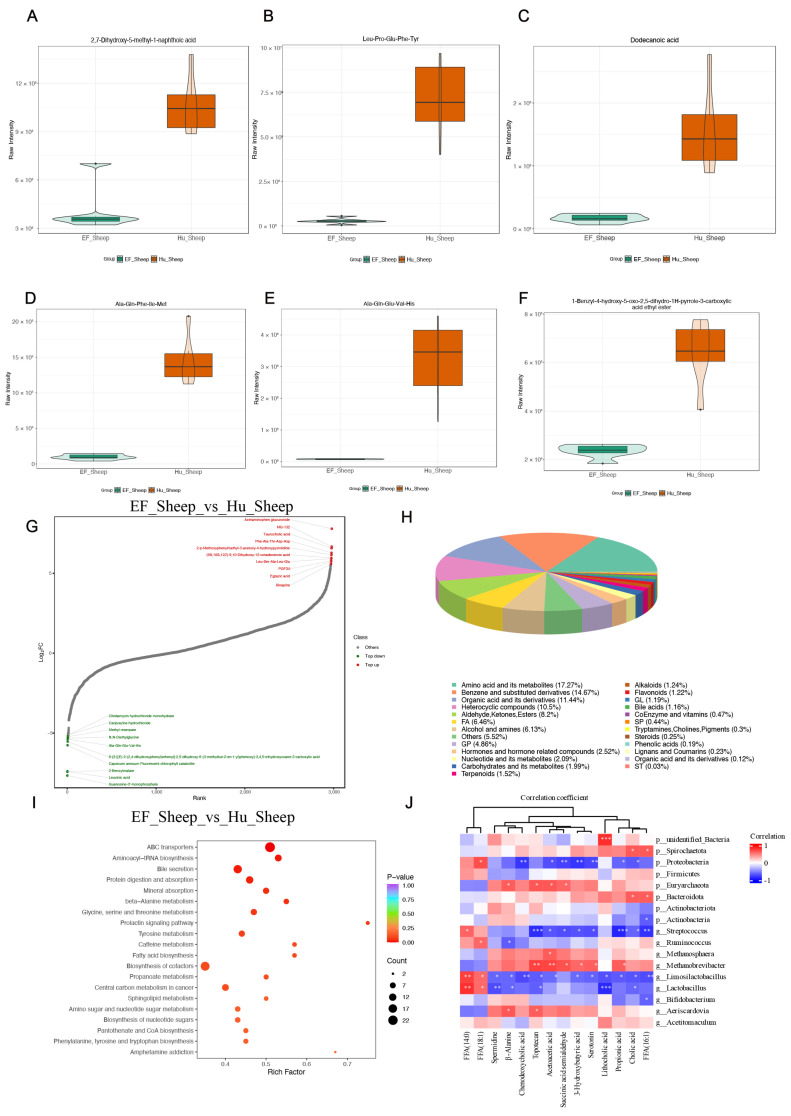
A comparison of intestinal metabolites between the Hu sheep and East Friesian sheep. (**A**–**F**) A violin diagram of metabolite change. (**G**) The dynamic distribution of the metabolite content differences and the classification of metabolite functions. (**H**) Each color in the pie chart represents a different classification, and the area represents the relative proportion of the metabolites. (**I**) Pathway enrichment analysis of differential metabolites between the Hu sheep and East Friesian sheep. (**J**) Analysis of the association of the microbiota with differentially abundant gut microbiota-related metabolites in the two groups of sheep by a partial Spearman’s correlation. * *p* < 0.05; ** *p* < 0.01; *** *p* < 0.001.

Compared to the EF sheep group, the violin plot visually shows that 2,7-Dihydroxy-5-methyl-1-naphthoic acid, Leu-Pro-Glu-Phe-Tyr, dodecanoic acid, Ala-Gln-Phe-Ile-Met, and Ala-Gln-Glu-Val-His were significantly upregulated in the Hu sheep group (Figure 4A–F). We selected the top 10 metabolites that were upregulated and downregulated in the Hu sheep and the EF sheep groups and found that most of the metabolites were amino acids of different types (Figure 4G).The classification of all the metabolites revealed that 17.27%, 11.44%, and 6.46% of the metabolites were amino acids and their metabolites, organic acids and their derivatives, and agents in fatty acid biosynthesis, respectively (Figure 4H). We utilized KEGG enrichment analysis to determine the biological functions of the perturbed metabolites, and the findings were generally consistent with the functional data from the microbiome. The KEGG enrichment results showed that the differentially abundant metabolites were mainly enriched in the bile secretion, beta-alanine metabolism, tyrosine metabolism, fatty acid biosynthesis, propanoate metabolism, sphingolipid metabolism, and phenylalanine, tyrosine, and tryptophan biosynthesis in the Hu sheep compared to the EF sheep groups (Figure 4I). To visualize the connection between the microbes and metabolites, we utilized Spearman’s correlation coefficient. As shown in this figure, *Lactobacillus* and *Methanosphaera* were significantly positively correlated with potential beneficial metabolites, such as amino acids and bile acids. These findings demonstrated that certain microbe changes interrupted normal intestinal activity and were linked to metabolic change (Figure 4J).

## 3. Discussion

Microbiota and their metabolites play important roles in gut health (various physiological functions), including nutrient absorption, energy storage, the maturation of host immune responses, the prevention of the proliferation of intestinal pathogens, and the response to or modification of specific drugs [26]. These are all associated with gut disease. For example, ruminants suffer mainly from gut microbial-related diseases, including diarrhea, enterotoxemia, and inflammatory bowel disease [27]. IBD is a gut microbial-related disease in which various gut microbial factors and abnormal immune responses result in abnormal host–microbial interactions [28]. Interestingly, the two breeds of sheep respond differently to these diseases, also known as different disease resistance [12]. In this study, we aimed to explore differences in the gut microbiota composition and metabolites between sheep with different genetic backgrounds and their impact on host physiology, primarily including disease resistance. It is worth noting that these differences are also related to the different production performances observed in these varieties.

The gut microbiota, including its richness and diversity, also play a key role in maintaining intestinal homeostasis and nutrient absorption [29]. In this process, host genetics impact the gut microbiota [30]. In essence, microbes can be thought as additional organs within the host organism [12]. Particularly, similarities can be drawn between our results and previous studies on pigs and cattle, which have identified different breeds of sheep with different microbial signatures [28]. Our survey showed no significant difference in the α diversity index between the two sheep breeds, indicating a similar richness and uniformity of microbial communities. As demonstrated by the OTU, Venn diagram, and PCA diagram, the β diversity of the ileum microorganisms was significantly different in the sheep from the two breeds. At the phylum level, the dominant intestinal flora, including *Firmicutes*, *Actinobacteriota*, *Bacteroidota*, and *unidentified_Bacteria*, remained statistically similar, consistent with previous studies of the gut flora in pigs, cows, and sheep [31]. The gut microbiota significantly affects the production performance of ruminants including calves, beef cattle, and dairy cows [21]. Evidence suggests that an increase in *Firmicutes* is associated with improved feed conversion in Holstein and Jersey cows [30]. The relative abundance of *Firmicutes* may be attributed to the difference in production performance between the two breeds, which is indicated by the correlation between *Firmicutes* and feed efficiency and immune responses [21]. This observation is consistent with other studies of the intestinal bacterial microbiota of ruminants including calves and beef cattle; *Lactobacillus* and *Bifidobacterium* have anti-inflammatory effects [32] and can influence behavior [33]. *Lactobacillus* can protect the repair of intestinal epithelial damage by pathogens and reduce the number of potential pathogens by increasing lactate [34]. *Lactobacillus* can express tryptophanase, the most important symbiont for metabolizing tryptophan [35]. *Lactobacillus* can affect inflammatory responses and immune regulation via Janus kinase/signal transduction and the activation of transcription signaling pathways [36]. There was a significant positive correlation between *Lactobacillus* abundance and feed utilization efficiency in pigs and cattle [21]. This observation implies that the Hu sheep may have better intestinal health conditions than the East Friesian sheep.

The differences observed in metabolites can be attributed to a variety of factors, including microbiota and diet, that work together to affect various physiological functions of sheep and pigs [21]. In this study, we used two groups of laboratory animals of similar ages and the same diet. Studies have shown that some metabolite changes may be related to microorganisms. The metabolite changes produced provide insights into the differences between the two breeds [28]. Microbial metabolites can interact with host cells to influence immune and inflammatory responses [37]. Compared to the EF sheep group, 2,7-Dihydroxy-5-methyl-1-naphthoic acid, Leu-Pro-Glu-Phe-Tyr, Ala-Gln-Phe-Ile-Met, and Ala-Gln-Glu-Val-His were significantly upregulated in the Hu sheep group. 2,7-Dihydroxy-5-methyl-1-naphthoic acid is the main ingredient of anti-inflammatory and anti-tumor drugs [38]. Consistent with our study, increased levels of certain amino acids may help improve the efficiency of pigs and cattle using other amino acids [39]. The higher concentration of amino acid metabolites indicated that the sheep had a better protein metabolism ability [38]. Furthermore, amino acids are condensed by dehydration to form polypeptides. Leu-Pro-Glu-Phe-Tyr, Ala-Gln-Phe-Ile-Met, and Ala-Gln-Glu-Val-His are short peptides with anti-inflammatory and antioxidant properties. The peptides containing Try, Cys, and His showed significantly stronger antioxidant activities, while those without specific amino acids showed weaker antioxidant activities [39]. Therefore, the Hu sheep, with a higher amino acid content, may better support protein metabolism to maintain growth and health.

KEGG pathway enrichment showed that these metabolites are mainly related to protein digestion and absorption, beta-alanine metabolism, propanoate metabolism, tyrosine metabolism, and phenylalanine, tyrosine, and tryptophan metabolism. Trypto-phan, propanoate, and their related metabolites may improve colitis and maintain intestinal barrier stability by inhibiting the activation of actin regulatory proteins [40]. Short-chain fatty acids, as key mediators linking disease, nutrition, and the gut microbiota, are also influenced by various factors, including microbiota, age, and genetic background. Gut microbes cause the production of SCFAs by fermenting undigested carbohydrates, proteins, and enzymes [5]. Propionic acid is a short-chain fatty acid that can promote the production of defense peptides and maintain immune homeostasis, ultimately affecting productivity [18]. Specifically, the enriched levels of propionic acid in the Hu sheep compared to the EF sheep group, indicating an enhanced growth performance, were also associated with impaired growth and health, consistent with other studies. Moreover, these metabolites are related to nutrient metabolism, particularly lipid metabolism, including linoleic acid metabolism, steroid degradation, fatty acid biosynthesis, sphingolipid metabolism, and bile secretion. Increased levels of fatty acids in the ileum, which provide animal energy, are consistent with previous studies in pigs. The regulation of glycerophospholipid metabolism reduces the synthesis of proinflammatory factors [12]. Bile salts also play a role in maintaining the acid–base balance in the intestines and inhibiting inflammation [41]. Bile salts also inhibit the growth of harmful bacteria, which helps to maintain the balance of intestinal flora and prevent intestinal infections and other diseases [42]. Through the functional analysis of all microorganisms, we found that the microorganisms could affect various biological metabolic pathways, including disease, metabolism, and organismal systems. The main enrichment pathways were cell growth and death and infectious, viral, and immune diseases. Consistent with previous research, the change in microbes and metabolites could influence cell growth and death and immune-related diseases [41]. Different breeds of sheep may produce specific substances that can absorbed and utilized more efficiently by hosts [21]. It partly explains the higher microbe and metabolite levels found in the Hu sheep and East Friesian sheep in this study. We hypothesize that various beneficial microbes and metabolites may interact with each other to modulate the immune response and ultimately affect disease resistance. This could play a key role in shaping the differences in the production performance between the two breeds of sheep. Therefore, the mechanisms by which microbes and metabolites may lead to differences in disease resistance remain to be investigated.

Several studies have shown that host genetic and environmental factors can affect the composition and function of gut microbes [37,43]. We first determined whether the ileal microbiota and metabolites of the two breeds of sheep differed and could produce different disease resistances. In this study, both breeds of sheep were kept on the same diet in different parts of the same sheepfold for a month, with only metal guardrails separating the breeds, allowing for a significant sharing of the environmental microbiota. Therefore, the environmental factors of these breeds were the same, and the microbial composition could be compared using 16s rRNA and nontargeted metabolomics. Using sheep with genetically distinct phenotypes, we revealed breed-associated variation in the small intestinal microbiota, characterized by a higher abundance of disease-resistant *lactobacillus* bacteria in the Hu sheep compared to the East Friesian sheep. In addition, we found for the first time that differences between two sheep breeds’ gut microbes led to significant alterations in amino acids, fatty acids, and bile acids. The Hu sheep were highly resistant to intestinal diseases, possibly because *Lactobacillus* and *Bifidobacterium* secreted the lactic acid to break down and metabolize bile salts. These microbes play a key role in repairing and protecting the gut. Through the functional analysis of all microorganisms, we found that the microorganisms could affect biological metabolic pathways, including cellular processes, environmental information processing, and disease, metabolism, and organismal systems in the primary functional classification. Therefore, our results hypothesize that the beneficial microbiota, amino acids, fatty acids, and bile acids have great potential for explaining the selective performance between the Hu sheep and East Friesian sheep. Disease resistance is the main reason for the difference between the two breeds of sheep in terms of performance. Through sequencing analysis, we found that the microorganisms and metabolites were significantly different. However, they may have affected the disease-resistant phenotype of the sheep, and the exact mechanism requires further experimental research and discovery, such as that using transcriptomics and metagenomics. This study provides new references and new ideas for improving the production performance, breeding, disease prevention, and treatment of East Friesian sheep.

## 4. Materials and Methods

### 4.1. Experimental Design and Methods

#### 4.1.1. Design of Animal Experiments

We used 6 healthy Hu sheep and 6 East Friesian sheep (purchased from the experimental farm of Inner Mongolia Leke Biotechnology Co., Ltd., Hohhot, Inner Mongolia Autonomous Region, China), with no documented disease or risk of disease during the two months. In pre-clinical animal studies, 6 animals are used in experimental designs as a sample size for each group, and the results are still statistically significant when the effect is large and the measurement accuracy is high [44]. A health check by a veterinarian ensured that all sheep complied with the laboratory requirements, including regarding their body weight, body temperature, appetite and water intake, behavior and activity levels, skin and coat condition, fecal condition, and urine condition [10]. The weight range was 15 kg ± 3 kg, and all sheep were in the same physiological state and were kept separately in sheds with troughs. The sheep were fed twice a day at 8 o’clock in the morning and 6 o’clock in the evening and were free to draw water. All sheep were free of antibiotics and probiotics, and all animals were males around 2 months of age, were fed hay and concentrate, and had free access to drinking water and inorganic mineral salts. All the sheep were randomly fed the same lamb concentrate supplement (purchased from Inner Mongolia Mengtai Land Biotechnology Development Co., Ltd. Hohhot, Inner Mongolia Autonomous Region, China) (Supplementary Materials) for over two weeks, and the feed ration composition adhered to the nutritional requirements outlined in the National Research Council (NRC) guidelines from 2012 [21].

#### 4.1.2. Conditions for Animals and Experiments

Native to China, Hu sheep (*Ovis aries*) are renowned for their ability to adapt to challenging conditions and their comparatively high meat-production efficiency. The European breed of East Friesian sheep is well known for its efficient growth traits and excellent milk production [21]. At the (Inner Mongolia Leke Biotechnology Co., Ltd.), both breeds were kept in a regulated setting. A temperature of 20–22 °C and a relative humidity of 50–60% were maintained. There was enough room and the lighting schedule was adjusted to a 12 h light/dark cycle, and the stocking density was kept at [0.5–1] sheep per square meter [8].

#### 4.1.3. Sample Collection Procedure

Samples of ileal content were taken from every sheep after an adaptation period of a certain amount of time, such as eight weeks. The sheep were fasted for 12 h before the samples were collected. Direct samples were taken from the ileum, placed on ice, and brought to the lab. Samples were gathered from animals that were healthy and free of disease to prevent contamination. The uniform sampling and collection of ileal contents from the sheep was performed using sterile longwall gloves (with new longwall gloves each time). All samples were distributed into 5 mL sterile freezable tubes and immediately stored in liquid nitrogen tanks until DNA was extracted. Each group of six was labeled as Hu sheep (Hu sheep) and East Friesian sheep (EF sheep) [22]. All animal experiments were performed by the National Research Council Guide for the Care and Use of Laboratory Animals and were approved by the Institutional Animal Care and Use Committee at Inner Mongolia University, China. The approval number is NMGDX 2022-0003.

### 4.2. DNA Extraction and 16S rRNA Gene Sequencing

The ileal contents of the Hu sheep and East Friesian sheep were sequenced for microbial and metabolite analysis. The sequencing was entrusted to Wuhan Maiwei Metabolic Biotechnology Co., Ltd. (Wuhan, China). All study data were calculated based on previously published research [45]. For the 16S rRNA gene amplification, each sample was subjected to PCR amplification in triplicate (biological replicates), and the amplicons were pooled before sequencing [45]. To ensure the accuracy and reliability of the amplification process, each PCR reaction was performed in technical duplicates, and the resulting products were verified for purity and concentration by agarose gel electrophoresis. The samples were extracted using the CTAB approach, and this was followed by the assessment of DNA purity and concentration by agarose gel electrophoresis. A suitable amount of sample DNA was transferred to a centrifuge tube and diluted to 1 ng/μL with sterile water. Using diluted genomic DNA as a template and selecting specific sequencing regions, barcoded primers were employed with the Phusion High Fidelity PCR Master Mix with GC Buffer from New England Biolabs^®^ (Ipswich, MA, USA), integrating efficient high-fidelity enzymes to guarantee amplification efficiency and precision. The primer binding sequences 341F (CCTAYGGRBGCACAG) and 806R (GGACTACN-NGGGTATCTAAT) were utilized to amplify the V3-V4 region of the 16S rDNA gene in bacteria; then, this was followed by the preparation of an amplicon pool for sequencing. Library creation and machine sequencing utilized the TruSeq^®^ DNA PCR Free Sample Preparation Kit for library construction. The Qubit (ThermoFisher Scientific, Waltham, MA, USA) and Agilent Bioanalyzer 2100 (Agilent Technologies Inc., Santa Clara, CA, USA) were utilized for library construction and q-PCR quantification. The samples were sequenced on a NovaSeq 6000, (San Diego, CA, USA).

### 4.3. Untargeted Analysis of Metabolites

The chromatographic separation was carried out on a Waters Acquity UPLC HSS T3 (Milford, MA, USA) column using a gradient of 0.1% formic acid in water and acetonitrile, with a flow rate of 0.3 mL/min. The samples were analyzed on a SCIEX TripleTOF 6600+ mass spectrometer (Framingham, MA, USA) in both positive and negative ESI modes, with a source temperature of 600 °C, ion spray voltages of 5500 V (positive) and −4500 V (negative), and a collision energy of 30 eV. The scan range was *m*/*z* 100–1500, and metabolites were identified using the KEGG database based on the *m*/*z* values [45]. Each sample was analyzed in triplicate biological replicates, with technical replicates included for PCR to ensure reproducibility. Metabolites were identified using the KEGG database, with a minimum identification confidence of 80% based on the *m*/*z* values and MS/MS fragmentation patterns.

### 4.4. Sequencing Data Analysis

#### 4.4.1. Sequencing Data Processing

The data for each sample were extracted from the offline dataset according to the barcode sequence and PCR amplification primer sequence, followed by a comparison of the barcode and primer sequences. Employing fastp (v0.22.0, https://github.com/OpenGene/fastp (accessed on 10 October 2024)), the initial reads were subjected to a filtering process to obtain high-quality reads as follows: We automatically identified and eliminated joint sequences. We discarded reads containing 15 or more N bases. We excluded reads with low-quality bases (mass value ≤ 20) constituting over 50%. We removed those with an average mass below 20 within a 4-base window. We eliminated polyG sequences at the terminus. And, we rejected reads shorter than 150 bp. High-quality dual-end reads were acquired via FLASH (v1.2.11, http://ccb.jhu.edu/software/FLASH/ (accessed on 10 October 2024)) to generate high-quality tag data (clean tags). The tag sequences were acquired via research (v2.22.1); the chimeric sequences were analyzed and identified using the species annotation database (https://github.com/torognes/vsearch (accessed on 10 October 2024)). The chimeric sequences were eventually removed to produce the final effective tags.

#### 4.4.2. Alpha Diversity Analysis

The Chao1, Shannon, Simpson, ACE, and PD-whole-tree indices were computed using the photoseq (v1.40.0) and vegan (v2.6.2) packages in R software (v4.2.0). R software (v4.2.0) was utilized to generate dilution, rank abundance, and species accumulation curves, while intergroup difference analysis of alpha diversity indices was conducted using R software. The investigation of intergroup variations in the alpha diversity index was performed utilizing both parametric and nonparametric methods.

#### 4.4.3. Beta Diversity Analysis

A comparative analysis of diversity was carried out the photoseq (v1.40.0) package in R software (v4.2.0) for calculating the UniFrac distance and developing a UPGMA sample clustering tree. The R software (version 4.2.0) was used to generate PCA and NMDS diagrams. PCA was conducted using the R software stats package, while PCoA and NMDS analyses were executed with the R software photoseq (v1.40.0) package. R software was used for intergroup analysis of the differences in the beta diversity index, and parametric and nonparametric tests were conducted.

#### 4.4.4. Linear Discriminant Analysis Effect Size

The differential microbiological analysis was performed via LEfSe [46]. The outcomes were subsequently compared across the two varieties to ascertain any similar ileal microbial indicators present in both. The procedures for the LEfSe analysis were as follows. LEfSe analysis was conducted using LEfSe (v1.1.2) software, employing a default LDA score filtering threshold of 4. A meta-analysis was conducted using Mothur software (v1.48.0) to execute intergroup permutation tests across many taxonomic levels (phylum, class, order, family, genus, and species) to derive *p*-values. The Benjamin and Hochberg method was employed to adjust the *p*-values and derive q-values. Anosim, MRPP, and Adonis analyses were performed with the Anosim, MRPP, and Adonis functions from the R vegan package, respectively [45].

The alpha diversity indicated the application of the indices using the vegan and photoseq packages (Chao1 and Shannon). Additionally, it made clear which statistical Kruskal–Wallis test was applied for intergroup comparisons. The beta diversity indicated the application of visualization methods (PCA and NMDS) and UniFrac distances. Additionally, it described the statistical techniques (Adonis, Anosim, and MRPP) that were employed to evaluate variations in the beta diversity. The differential abundance explained how to apply the Benjamani–Hochberg FDR for multiple testing corrections and how to use the LEfSe and Metastats for microbiome analysis [10].

## 5. Conclusions

The two breeds of sheep respond differently to diseases, including intestinal and respiratory diseases, which is also known as different disease resistances. In this experiment, we found that both breeds of sheep had differential microbes and metabolites. The relative abundances of *Lactobacillus* and *Bifidobacterium* in the Hu sheep group were higher, and these can affect inflammatory responses and immune regulation, thus benefiting sheep. The Hu sheep group had more 2,7-Dihydroxy-5-methyl-1-naphthoic acid, Leu-Pro-Glu-Phe-Tyr, Ala-Gln-Phe-Ile-Met, and Ala-Gln-Glu-Val-His, and these can affect the gut microbiota, the intestinal barrier, the immune response, and amino acid metabolism, ultimately affecting productivity. Our results suggest that ileal microbes and metabolites may be among the important factors affecting host disease resistance in these two breeds of sheep. These findings provide new insights into the underlying mechanisms of differences in the production traits between the Hu sheep and East Friesian sheep from the perspective of ileal microbiology.

## Data Availability

The datasets used during the current study are available from the corresponding author upon reasonable request.

## Supplementary Materials

The following supporting information can be downloaded at https://www.mdpi.com/article/10.3390/ijms252413267/s1.
